# Supplementary material for: MetaQTL: a package of new computational methods for the meta-analysis of QTL mapping experiments
Source: BMC Bioinformatics. 2007 Feb 8;8:49. doi: 10.1186/1471-2105-8-49 (PMC1808479; doi:10.1186/1471-2105-8-49)
Supplement: Additional File 7 — MetaQTL Package : jar file and tutorial. This Zip archive contains both the MetaQTL JAR file and the files of the tutorial. [file 1471-2105-8-49-S7.zip › org.inra.metaqtl/doc/org/metaqtl/class-use/EMResult.html]

Uses of Class org.metaqtl.EMResult


|  |  |  |
| --- | --- | --- |
| **Overview** \| **Package** \| **Class** \| **Use** \| **Tree** \| **Deprecated** \| **Index** \| **Help** | |  |
| PREV   NEXT | **FRAMES**    **NO FRAMES**     **All Classes** |  |


---


## **Uses of Class org.metaqtl.EMResult**

| Packages that use EMResult | |
| --- | --- |
| **org.metaqtl** |  |
| **org.metaqtl.adapter** |  |
| **org.metaqtl.algo** |  |
| **org.metaqtl.factory** |  |

Uses of EMResult in org.metaqtl

| Fields in org.metaqtl declared as EMResult | |
| --- | --- |
| `EMResult[]` | `MetaQtlResult.clusterings`             The meta qtl clusterings. |

| Methods in org.metaqtl that return EMResult | |
| --- | --- |
| `EMResult` | `MetaQtlResult.getBestClustering(int criterion)`             This methods returns the best models for each criterion defined to select the optimal number of clusters per trait. |
| `static EMResult` | `EMCriteria.getBestResult(EMResult[] clusterings, int criterion)`             For the given criterion `criterion` this methods looks into the array of `EMResult` and find the model which is optimal. |
| `static EMResult` | `EMCriteria.getBestResult(EMResult[] clusterings, int n, int criterion)` |
| `static EMResult` | `EMCriteria.getBestResult(EMResult[] clusterings, int n, java.lang.String criterion)` |
| `static EMResult` | `EMCriteria.getBestResult(EMResult[] clusterings, java.lang.String criterion)` |
| `EMResult` | `MetaQtlResult.getClustering(int k)`             Returns the Qtl clustering result with `k` clusters. |

| Methods in org.metaqtl with parameters of type EMResult | |
| --- | --- |
| `static void` | `EMResult.copy(EMResult dest, EMResult src)` |
| `static EMResult` | `EMCriteria.getBestResult(EMResult[] clusterings, int criterion)`             For the given criterion `criterion` this methods looks into the array of `EMResult` and find the model which is optimal. |
| `static EMResult` | `EMCriteria.getBestResult(EMResult[] clusterings, int n, int criterion)` |
| `static EMResult` | `EMCriteria.getBestResult(EMResult[] clusterings, int n, java.lang.String criterion)` |
| `static EMResult` | `EMCriteria.getBestResult(EMResult[] clusterings, java.lang.String criterion)` |
| `double[]` | `MetaQtlResult.getUSD(EMResult best, int kmin, int kmax, java.lang.String criterion)` |
| `void` | `MetaQtlResult.setClusterings(EMResult[] results)` |

Uses of EMResult in org.metaqtl.adapter

| Methods in org.metaqtl.adapter with parameters of type EMResult | |
| --- | --- |
| `static Qtl[]` | `QtlAdapter.adapt(EMResult result)`             From a result of a EM clustering returns the mixture components as an array of Qtl. |

Uses of EMResult in org.metaqtl.algo

| Methods in org.metaqtl.algo that return EMResult | |
| --- | --- |
| `static EMResult` | `EMAlgorithm.doEM(double[] x, double[] sd, int k, EMResult spoint)`             Apply the EM-Algorithm on the given data set where `x` is an array of observed value and `sd` an array of same size than `x` which stores the standard deviations of the observed values. |

| Methods in org.metaqtl.algo with parameters of type EMResult | |
| --- | --- |
| `static void` | `EMAlgorithm.computeCOV(double[] sd, EMResult theta)`             Computes the variance-covariance matrix of the mixture component estimates. |
| `static void` | `EMAlgorithm.computeDM(double[] x, double[] sd, double[] mu, EMResult theta)`             Computes the matrix of derivatives of the update function. |
| `static EMResult` | `EMAlgorithm.doEM(double[] x, double[] sd, int k, EMResult spoint)`             Apply the EM-Algorithm on the given data set where `x` is an array of observed value and `sd` an array of same size than `x` which stores the standard deviations of the observed values. |
| `static void` | `EMAlgorithm.emRate(EMResult theta, double[] mu, double[] pi)`             Compute the euclidean distance between the new parameters and the old ones to obtain a simple approximation of the EM convergence rate. |
| `static void` | `EMAlgorithm.eStep(double[] x, double[] sd, EMResult theta)`             The Expectation step : it just consists in updating the Z matrix, i.e the cluster membership probabilities. |
| `static void` | `EMAlgorithm.initEM(double[] x, double[] sd, EMResult theta, java.util.Random rng)`             Initializes the EM algorithm by randomly assigning observations to the clusters and do one M-Step to compute the first values of the parameters. |
| `static int` | `EMAlgorithm.iterate(double[] x, double[] sd, EMResult theta)`             This method performs one iteration of the EM-algorithm. |
| `static int` | `EMAlgorithm.mStep(double[] x, double[] sd, EMResult theta, double err)`             The Maximization step : here this step is straighforward and simple analytical formula are applied to obtain the new parameter estimates. |
| `static void` | `EMAlgorithm.updateLoglikelihood(double[] x, double[] sd, EMResult theta)`             Updates the loglikelihood value. |
| `static double[]` | `EMAlgorithm.updateMuVector(double[] x, double[] sd, EMResult theta)`             Updates the vector of mixture components and returns the new values. |
| `static double[]` | `EMAlgorithm.updatePiVector(EMResult theta)`             Updates the vector of mixture mixings and returns the new values. |
| `static void` | `EMAlgorithm.updateZMatrix(double[] x, double[] sd, EMResult theta)`             Updates the Z matrix. |

Uses of EMResult in org.metaqtl.factory

| Methods in org.metaqtl.factory that return EMResult | |
| --- | --- |
| `static EMResult` | `EMResultFactory.read(java.io.BufferedReader buffer)` |

| Methods in org.metaqtl.factory with parameters of type EMResult | |
| --- | --- |
| `static void` | `EMResultFactory.write(EMResult result, java.io.OutputStream stream)` |
| `static void` | `EMResultFactory.write(EMResult result, java.io.PrintWriter writer)` |

---


|  |  |  |
| --- | --- | --- |
| **Overview** \| **Package** \| **Class** \| **Use** \| **Tree** \| **Deprecated** \| **Index** \| **Help** | |  |
| PREV   NEXT | **FRAMES**    **NO FRAMES**     **All Classes** |  |


---
